# Alpha-Lipoic Acid Supplementation Restores Early Age-Related Sensory and Endothelial Dysfunction in the Skin

**DOI:** 10.3390/biomedicines10112887

**Published:** 2022-11-10

**Authors:** Anne-France de Bengy, Johanna Decorps, Lisa S. Martin, Aurélie Pagnon, Fabien P. Chevalier, Dominique Sigaudo-Roussel, Bérengère Fromy

**Affiliations:** 1 AF BioCell, 59 Avenue du Point du Jour, 69005 Lyon, France; 2 CNRS, LBTI UMR5305, Univ. Lyon, Université Claude Bernard Lyon 1, 7 Passage du Vercors, CEDEX 7, 69367 Lyon, France; 3 Novotec Labs, ZAC du Chêne, Europarc, 11 rue Edison, 69500 Bron, France

**Keywords:** skin aging, alpha lipoic acid, Brown Norway, Wistar, microcirculation, blood flow, pressure-induced vasodilation, skin resistance to pressure

## Abstract

Many changes characterize skin aging, and the resulting dysfunctions still constitute a real challenge for our society. The aim of this study was to compare the skin aging of two rat strains, Wistar and Brown Norway (BN), considered as “poorly aging” and “healthy aging” models, respectively, and to assess the effect of alpha-lipoic acid (LPA), especially on skin microcirculation. To this purpose, various skin characteristics were studied at 6, 12, and 24 months and compared to the results of LPA treatment performed at 12 or 24 months. Skin aging occurred in both strains, but we showed an early occurrence of different age-related disorders in the Wistar strain compared to BN strain, especially regarding weight gain, glycemia dysregulation, basal skin perfusion, endothelial function, and skin resistance to low pressure. LPA treatment tended to improve skin resistance to low pressure in BN but not in Wistar despite the improvement of basal skin perfusion, endothelial function, and skin sensory sensitivity. Overall, this study confirmed the healthier aging of BN compared to Wistar strain and the positive effect of LPA on both general state and skin microcirculation.

## 1. Introduction

Many physiological changes are correlated with aging, including altered vascular signaling and concomitant endothelial dysfunction. In the skin, endothelial dysfunction has been well described in humans [1,2,3] and in rodents [4], with significant interindividual variation in the age of onset [5]. In addition to indicating cardiovascular pathogenesis [6], it also favors skin disorders such as pressure injury, whose prevalence remains too high in the aging population [7,8,9]. A decline in sensory nerve function and a disturbance in its modulation of microvascular blood flow (the sensory neurovascular system) were also reported with aging, worsening the risk for pressure injury [8] and delaying tissue repair [10].

In healthy skin, a local application of low pressure induces an increase in cutaneous blood flow, called pressure-induced vasodilation (PIV) [11]. This mechanism results from the interaction of sensory nerve endings and vascular endothelium of skin vessels. Some vasodilator peptides such as calcitonin gene-related peptide (CGRP) secreted by capsaicin-sensitive nerve fibers can stimulate endothelial cells and induce the secretion of paracrine factors such as nitric oxide (NO) or prostaglandins that stimulate the relaxation of the smooth muscle cells surrounding blood vessels and, thus, vasodilation [12]. Individuals without a normal PIV response show an early decrease of cutaneous blood flow to the application of very low pressures, reflecting a vascular fragility of the skin associated with higher risk of pressure ulcer formation [13]. With aging, PIV ability was found reduced in 60 year old human and 24 month old mouse skin compared to younger adults, and this reduction was associated with a dysfunction of endothelial cells and/or sensory perception [1,4]. The excess of reactive oxygen species (ROS) production related to endogenous antioxidant defenses is a primary mechanism contributing to reduced NO bioavailability, inducing vascular dysfunction with age [14,15,16]. 

Lipoic acid (LPA) has been shown to have many positive effects on fragile skin. A topical application for 12 weeks with a cream containing 5% LPA was reported to improve significantly clinical characteristics related to photoaging of human facial skin, especially skin roughness [17]. Effective epidermal thickening has been confirmed in human skin after a treatment containing LPA applied for 6 months [18]. These positive effects of LPA reported in humans were also demonstrated by several animal studies [19,20,21], thus highlighting the role of LPA in improving several skin pathological conditions. Moreover, LPA is known for his strong antioxidant effects [22]. In vivo and in vitro in monocyte/macrophage-like cells, LPA and dihydro-lipoic acid (DHLA) have been reported in mice to inhibit effectively NO and prostaglandin E2 formation induced by lipopolysaccharide (LPS) in correlation with NO synthase (NOS) and cyclooxygenase-2 (COX2) inhibition [23]. In addition, LPA treatment prevented the reduction of skin PIV in short-term diabetic mice by limiting oxidative stress and by preserving endothelial function (i.e. preserving NO production and/or preventing NO destruction) [24]. However, the ability of LPA to improve skin homeostasis and limit vascular dysfunction during aging remains unexplored. 

Chronological aging is an inexorable process, but it can also be accelerated with the development of various pathologies depending on the individual. This led us to focus on the effect of LPA on cutaneous microcirculation during aging in two rat strains, particularly considering endothelial function and PIV abilities. The Brown Norway (BN) strain is considered as a “healthy aging model” since it is known to survive relatively disease-free until a very old age [25]. In contrast, the Wistar strain is reported as a “poorly aging model” since it develops more diseases with aging. To observe the progressive evolution during skin aging, we studied both strains at three different ages: young adult (6 months), middle-aged (12 months), and old rats (24 months) [26].

Age-related disorders (weight gain, glycemia dysregulation, cutaneous endothelial dysfunction, and inability of the skin to resist low pressures) were more pronounced or earlier in the Wistar strain than in the BN strain. Despite some beneficial effects on the general body characteristics, NO bioavailability, and cutaneous sensory thresholds, LPA treatment failed to restore skin resistance in the Wistar strain, even in middle-aged rats. In contrast, LPA tended to improve skin resistance to pressure in middle-aged BN rats, delaying the decrease in skin blood flow to higher pressures. 

## 2. Materials and Methods

### 2.1. Animals

#### 2.1.1. Animal Experiments

The experiments were performed in Wistar and BN rat strains (Harlan, Gannat, France) aged 6, 12, and 24 months. To avoid inter-sex variability, only male rats were used. The animals were housed with ad libitum access to food and water, in a regulated environment with a constant temperature of 24 °C and a day/night cycle of 12 h/12 h. All experiments were conducted in accordance with European Union recommendations for animal experimentation and approved by the Animal Experimentation Committee of the University Claude Bernard Lyon 1 (protocol n^o^ BH2011-38). Special effort was made to minimize the number, as well as the stress and suffering, of rats used in this study. Before experimentation, all animals were acclimatized for at least 1 week. 

#### 2.1.2. LPA Treatment

Separate groups of rats aged 12 or 24 months of both strains were fed a diet supplemented with 0.5% alpha-lipoic acid (LPA) for 4 weeks prior to the experiments to assessing its effect on age-related changes compared to untreated young (6 month old) and untreated age-matched rats in all parameters described below.

### 2.2. General State

The animal weight and the systolic arterial blood pressure were assessed for each animal, and a glucose tolerance test was performed after a 12 h fast. Glucose (30%; 0.7 mL/100 g) was administered by gavage at time 0. Blood was collected from the tail vein at different times, including 2.5 h after feeding, and the blood glucose concentration was measured using a glucometer (Accu-Check^®^ Active; Roche, Lyon, France).

### 2.3. Microvascular Experiments

Measurement of skin blood flow was performed using noninvasive laser Doppler techniques under well-calibrated conditions (skin temperature, depth of anesthesia, and animal stress) as in previous studies with rats [27,28,29].

#### 2.3.1. Animal Preparation

First, 48 h before each experiment, the rats previously anesthetized using a volatile anesthetic (isoflurance) were shaved and depilated on the back and thighs using Veet^®^ depilatory cream (Reckitt Benckiser, Massy, France). 

On the day of the experiment, the animal was anesthetized with an intraperitoneal injection of sodium thiopental at 65 mg/kg (Nesdonal; Merial, Lyon, France). After testing the ocular reflex and pinching the paw to control anesthesia depth, the animal was placed prone in an environmentally controlled incubator (MMS, Chelles, France) maintaining the skin temperature at 35.0 ± 0.5 °C. This cutaneous temperature was measured using a thermocouple (Physitemp BAT-12, Physitem instruments, Clifton, NJ, USA). Systolic arterial blood pressure was recorded noninvasively at the tail (XBP-1000, Kent Scientific, Torrington, CT, USA) before and at the end of the experiment to check its stability regarding the skin blood flow recording.

At least 10 min of observation was implemented for the stabilization of arterial blood pressure and skin temperature before starting the cutaneous microvascular measurements. 

#### 2.3.2. Skin Resistance to Low-Pressure Application (PIV)

PIV was assessed using a weighbridge adapted to hold a laser Doppler probe (PF801, Periflux, Perimed AB, Järfälla, Sweden) as previously described [27]. After a 1 min control period, local and regularly increasing pressure was applied at 11.1 Pa·s^−1^ for 20 min on the hairless thigh skin of the anesthetized rat. The laser Doppler flow (LDF) signals were averaged every 30 s to reduce the instantaneous variability of the signals due to vasomotion.

**Figure 1:**
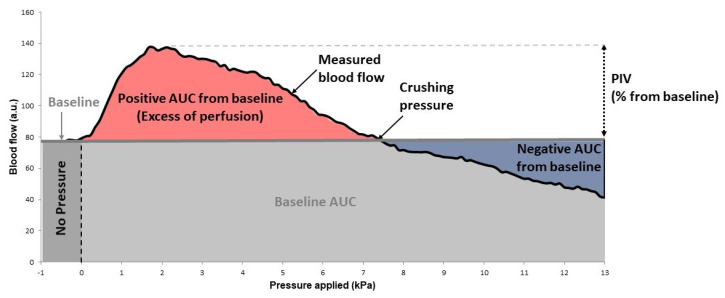
Evolution of the blood flow under pressure application and definition of PIV, along with crushing pressure, positive and negative AUC from baseline.

In each condition, a significant increase in blood flow compared to the baseline value (calculated as the average over the 1 min control period prior to the onset of local pressure increase) was validated using statistical analysis (Kruskal–Wallis test). PIV was reported as the maximal percentage increase in skin blood flow from baseline in response to local pressure application. As pressure continues to increase, skin blood flow begins to decrease, reaching a level under the baseline value at a crushing pressure determined from the mean curve (Figure 1). From the curves representing the variations in skin blood flow in response to local application of increasing pressure, we determined the positive area under the curve from baseline (positive AUC from baseline) representing the whole excess of perfusion until the crushing pressure was reached (Figure 1). We also determined the negative AUC from baseline corresponding to the deficit of perfusion induced by pressures higher than the crushing pressure (Figure 1). 

#### 2.3.3. Endothelium Dependent or Independent Vasodilation

To assess the functional integrity of skin microcirculation, the endothelium-dependent and -independent vasodilator responses were used to evaluate vascular endothelium or smooth muscle function, respectively. The endothelium-independent vasodilation was induced in the cutaneous microcirculation by iontophoretic delivery of 67 mM sodium nitroprusside (SNP) using cathodal current (100 μA) for 30 s, while the endothelium-dependent vasodilation was induced by iontophoretic delivery of 5.5 mM acetylcholine (Ach) using anodal current (100 μA) for 20 s. The iontophoretic delivery was performed using a laser Doppler probe (481-1, Perimed AB, Järfälla, Sweden) positioned on the hairless back skin of the anesthetized rat. This probe also allowed for cutaneous blood flow recording.

Data collection began with a 1 min basal period prior to the onset of current application and was continuously recorded for 16 min. Preliminary studies were performed to verify that there was no effect of iontophoretic delivery of deionized water with the use of anodal and cathodal currents of 100 μA for 20 s and 30 s, respectively.

#### 2.3.4. Contribution of NOS and COX Pathways in Ach-Mediated Vasodilation

For each condition, Ach iontophoresis was performed 10 min following the cutaneous injection of a specific inhibitor of constitutive NOS (Nω-nitro-l-arginine, LNNA, 0.1 mL, 20 mg·kg^−1^, Sigma, St Quentin Fallavier, France), either alone or in combination with a nonspecific inhibitor of COX (indomethacine, INDO, 0.1 mL, 5 mg·kg^−1^, Sigma, St Quentin Fallavier, France). Control rats received saline injection. 

### 2.4. Skin Sensory Nerve Fiber Sensitivity Analysis

Stimulus-evoked behavioral methods were used in conscious rats to elicit a withdrawal response in order to evaluate thermal and mechanical sensory sensitivities [30,31].

#### 2.4.1. Tail Flick Test

The conscious rats were held manually, and a cloth was used to cover their head and keep them calm during the experiment. Their tail (about 1.5 cm from the tip) was placed under a radiant heat source produced by a halogen lamp of a device (2TC series 8 Model, ITT Inc. Life science, CA, USA) previously calibrated for 30 s to deliver 50 W of heat. The heating rate was 1.3 °C/s, and the delay in tail removal was measured. A cutoff time of 10 s was imposed to prevent tissue damage. Five measurements of the tail withdrawal latency were taken and averaged for each rat to determine the nociceptive thermal threshold for the animal. 

#### 2.4.2. Von Frey Test

The mechanical threshold for hind paw withdrawal was determined using an electronic Von Frey anesthesiometer (Almemo 2390-5 Ahlborn, 2TC Inc., Woodland Hill, CA, USA). Rats were placed in clear Plexiglass boxes on an elevated mesh screen. Mechanical pressure was applied to the plantar surface of the right and left hind paws in a series of ascending forces using a rigid blunt tip. Five measurements of the mechanical threshold were taken and averaged for each rat.

#### 2.4.3. Randall–Selitto Test (Mechanical Noxious Stimuli)

The rat hind paw withdrawal reflex to nociceptive pressure stimulation was determined using the Randall–Selitto assay. When the rat vocalized or withdrew its paw to respond to uniformly increasing pressure, the sinking threshold of the animal was recorded in grams. Five measurements of the mechanical nociceptive threshold were taken and averaged for each rat. 

### 2.5. Statistical Analyses

Data are expressed as means ± SEM. A two-way ANOVA followed by a Sidak multiple-comparison test was used to determine the statistical difference between the two strains and the three stages of aging (6, 12, and 24 months). According to data normality, a one-way ANOVA or Kruskal–Wallis test followed by Tukey’s or Dunn’s multiple comparison test, respectively, were used to determine LPA effects within each strain. A *p*-value less than 0.05 was regarded as statistically significant.

## 3. Results

### 3.1. LPA Limited Weight Gain and Improved Glycemia Regulation of “Healthy Aging” BN strain and “Poorly Aging” Wistar Strain

**Figure 2:**
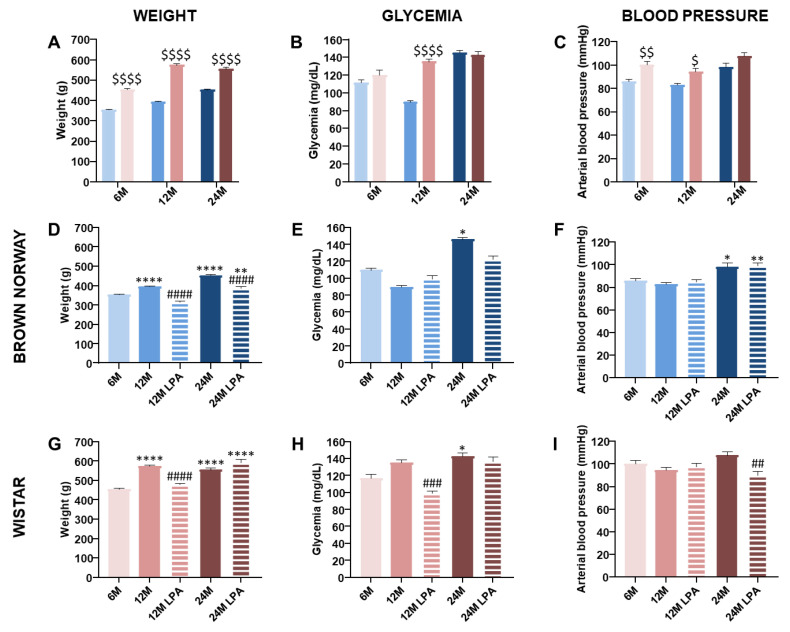
Evolution of the animal weight (**A**,**D**,**G**), glycemia 2.5 h after glucose absorption (**B**,**E**,**H**), and arterial blood pressure (**C**,**F**,**I**) over time with (hatched bars) or without (solid bars) LPA treatment. The results were obtained for both BN (in blue) and Wistar (in pink) strains for 6 month old (6 M), 12 month old (12 M), and 24 month old (24 M) rats previously fed a diet supplemented with α-lipoic acid (0.5%) for 4 weeks (LPA) or not. The results were compared between the two strains (**A**–**C**) or within the BN strain (**D**–**F**) or Wistar strain (**G**–**I**). Error bars represent the standard error of the mean. Statistical analyses were performed revealing significant differences between the two strains for each age group ($ *p* < 0.05; $$ *p* < 0.01; $$$$ *p* < 0.0001 vs. BN strain) or within each strain during skin aging when compared to the 6 month condition (* *p* < 0.05; ** *p* < 0.01; **** *p* < 0.0001) and after LPA treatment compared to age-matched untreated condition (## *p* < 0.01; ### *p* < 0.001; #### *p* < 0.0001).

As expected, all rats gained weight with aging, although BN rats were significantly lighter than Wistar rats regardless of age (Figure 2A). LPA treatment significantly reduced the weight gain at 12 months for BN and Wistar rats (Figure 2D,G). This effect was preserved at 24 months only for BN (Figure 2D).

Following glucose absorption, the blood glucose level of all 6 month old animals returned to basal value after 2.5 h, showing a similar ability to regulate glycemia at 6 months (Figure 2B). This ability was disturbed earlier in Wistar strain (Figure 2H) than in the BN strain (Figure 2E). Indeed, blood glucose level was higher 2.5 h post absorption in Wistar rats compared to BN rats at 12 months (Figure 2B), but no longer at 24 months. LPA treatment only improved this ability to regulate glycemia at 12 months in Wistar compared to age-matched untreated rats (Figure 2H). 

The systemic arterial blood pressure was higher in the Wistar strain than BN at 6 and 12 months (Figure 2C). Although values remained in the normal range of systemic arterial blood pressure, aging slightly increased this parameter, especially in 24 month old BN rats (+14% vs. 6 months) (Figure 2F). LPA treatment only decreased this parameter in 24 month old Wistar rats (Figure 2I). 

Overall, higher body weight and systolic arterial blood pressure, as well as earlier age-related effects on glycemia regulation, occurred in Wistar rats than in BN rats. These results are in line with previous studies suggesting that the BN strain survives relatively disease-free until very old age [25]. In the Wistar strain, our results highlight the ability of LPA to reduce the age-related weight gain and improve the ability to regulate glycemia only in middle-aged rats. In the BN strain, LPA was able to reduce the slight age-related weight gain at 12 and 24 months. In our models, aging induced only very limited effects on systolic arterial pressure, and LPA treatment only decreased this parameter in 24 month old Wistar rats. These results seem to indicate beneficial effects of LPA on moderate and/or short-term alterations during aging.

### 3.2. LPA Tended to Improve the Age-Related Alterations of Skin Ability to Resist to Low Pressures in Old BN but Not in Wistar Strain

In humans and rodents, a local application of increasing pressure on healthy skin induces an increase in skin blood flow reported as PIV [13].

**Figure 3:**
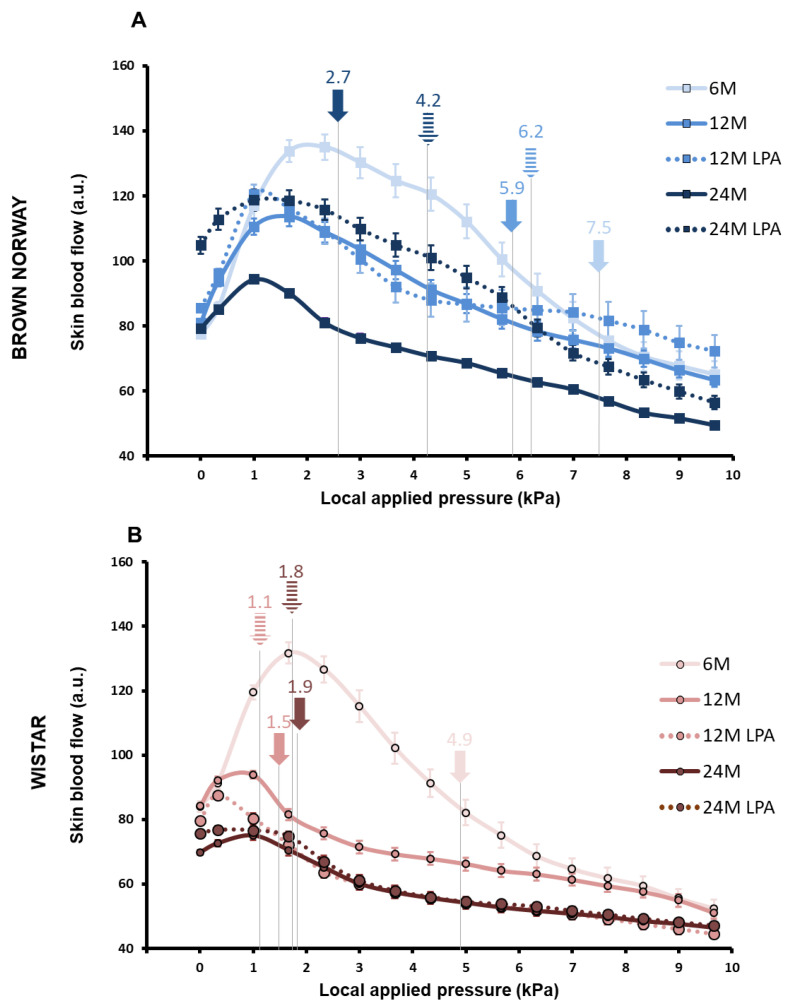
Skin blood flow changes in response to local external pressure application with (hatched lines) or without (solid lines) LPA treatment. The results were obtained for both BN (**A**) and Wistar (**B**) strains for 6 month old (6 M), 12 month old (12 M), and 24 month old (24 M) rats previously fed a diet supplemented with α-lipoic acid (0.5%) for 4 weeks (LPA) or not. In each case, the skin blood flow depending on the pressure applied was registered, and the crushing pressure means (numbers and arrows on the curve for each condition) were noted. Error bars represent the standard error of the mean.

**Figure 4:**
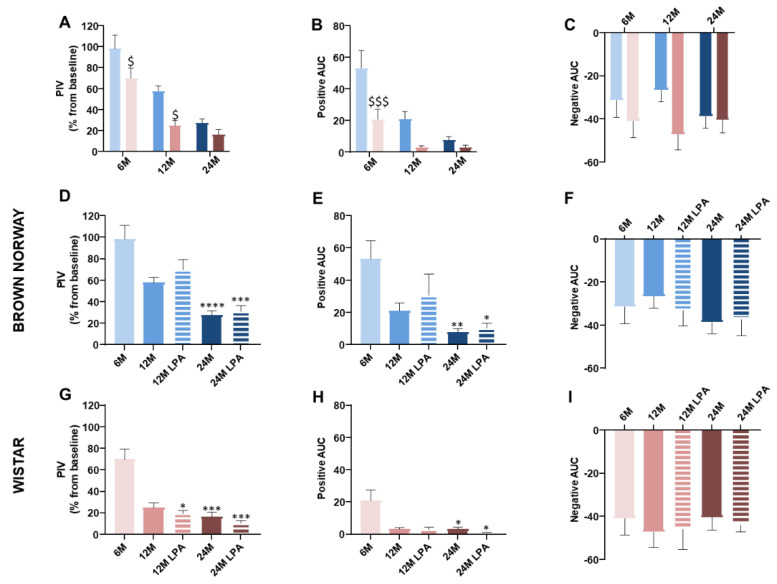
Skin ability to resist low pressure with (hatched lines/bars) or without (solid lines/bars) LPA treatment. The results were obtained for both BN (in blue) and Wistar (in pink) strains for 6 month old (6 M), 12 month old (12 M), and 24 month old (24 M) rats previously fed a diet supplemented with α-lipoic acid (0.5%) for 4 weeks (LPA) or not. The results were compared between the two strains (**A**–**C**) or within the BN strain (**D**–**F**) or Wistar strain (**G**–**I**). In addition to the skin blood flow changes in response to pressure application, the PIV (**D**,**G**), the positive AUC from baseline (**E**,**H**), and the negative AUC from baseline (**F**,**I**) were calculated for each strain in each condition and compared between the two strains (**A**–**C**). Error bars represent the standard error of the mean. Statistical analyses were performed revealing significant differences between the two strains for each age group ($ *p* < 0.05; $$$ *p* < 0.001 vs. BN strain) or within each strain during skin aging when compared to the 6 month condition (* *p* < 0.05; ** *p* < 0.01; *** *p* < 0.001; **** *p* < 0.0001).

By applying a local and progressively increasing pressure on skin in 6 month old rats, we confirmed the presence of PIV in both strains (Figure 3), although PIV was about 30% lower in Wistar compared to BN rats (Figure 4A). Interestingly, not only the PIV amplitude (Figure 4A) but also the mean crushing pressure (Figure 3) were lower in the 6 month old Wistar (4.9 kPa) compared to BN rats (7.5 kPa), resulting in a significantly lower excess of perfusion (defined as positive AUC) for 6 month old Wistar rats (Figure 4B). For pressures higher than the crushing pressure, skin blood flow further decreased in both strains, resulting in a similar deficit of perfusion (negative AUC) for 6 month old BN and Wistar rats (Figure 4C,F,I). This highlights a distinct response for very low pressures, rather than for higher pressures leading to ischemia.

As expected with aging [1,4], PIV was reduced in both strains (Figure 4A). However, we observed a gradual decrease in PIV in BN rats (−41% at 12 months and −72% at 24 months compared to 6 month old rats), while a drastic reduction occurred in Wistar from 12 months (−64% at 12 months and −77% at 24 months) (Figure 4D,G). Similar observations were made for positive AUC (Figure 4E,H). Consequently, the mean crushing pressure decreased progressively with aging in BN rats (5.9 kPa at 12 and 2.7 kPa at 24 months) (Figure 3A), while it decreased below 2 kPa from 12 months in Wistar rats (Figure 3B). Regardless of the age, PIV, positive AUC, and mean crushing pressure were lower in Wistar compared to BN rats, even if the difference disappeared at 24 months (Figure 4A,B). For pressures higher than the crushing pressure, aging did not have any significant effect on the negative AUC for both strains (Figure 4C). Altogether, these results suggest that skin resistance to low pressure decreased with skin aging for both strains but remained higher and decreased later for BN. This is in line with a healthier skin aging for the BN strain compared to Wistar, as previously reported on other parameters [25]. Nevertheless, the results also highlight the occurrence of age-related alterations of skin ability to resist to low pressures even in a “healthy aging” model such as BN strain. 

In BN rats, LPA treatment tended to improve PIV (+21%) and positive AUC (+44%) at 12 months compared to age-matched untreated batches, without reaching significance (Figure 4D,E). This positive effect was almost not observed at 24 months. However, the mean crushing pressure was increased by LPA treatment at 12 months (+5%) and 24 months (+56%) compared to age-matched untreated rats (Figure 3A). This suggests that LPA tended to improve age-related alterations in skin resistance to low pressures by delaying the decrease in skin blood flow from baseline for higher crushing pressure in BN strain. 

In contrast, the results obtained in LPA-treated Wistar rats looked very similar compared to untreated populations of the same age (Figure 4G–H). LPA treatment even tended to slightly decrease PIV and positive AUC. In addition, the mean crushing pressure was further decreased at 12 months (−27%) and 24 months (−5%) compared to age-matched untreated Wistar rats (Figure 3B). These results indicate that LPA was not able to improve skin resistance to pressure at all in Wistar strain, regardless of the age. For pressures higher than the crushing pressure, LPA treatment did not have any significant effect on both strains (Figure 4F,I). 

The succession of steps leading to PIV involves (i) pressure stimulation of specialized sensory nerve fibers, (ii) release of vasodilator neuropeptides such as calcitonin gene-related peptide (CGRP) from afferent sensory nerve endings in response to local pressure application, and (iii) liberation of vasodilator endothelial factors leading to vascular smooth muscle relaxation [11,12,13,27,32]. Because PIV requires intact functional integrity of the sensory nervous system, further experiments were conducted to measure the skin thermal threshold using the tail flick test, as well as the skin mechanical threshold using the Von Frey and Randall–Selitto tests [33,34,35,36,37]. 

### 3.3. LPA Improved Skin Sensory Nerve Sensitivity in Both Strains

**Figure 5:**
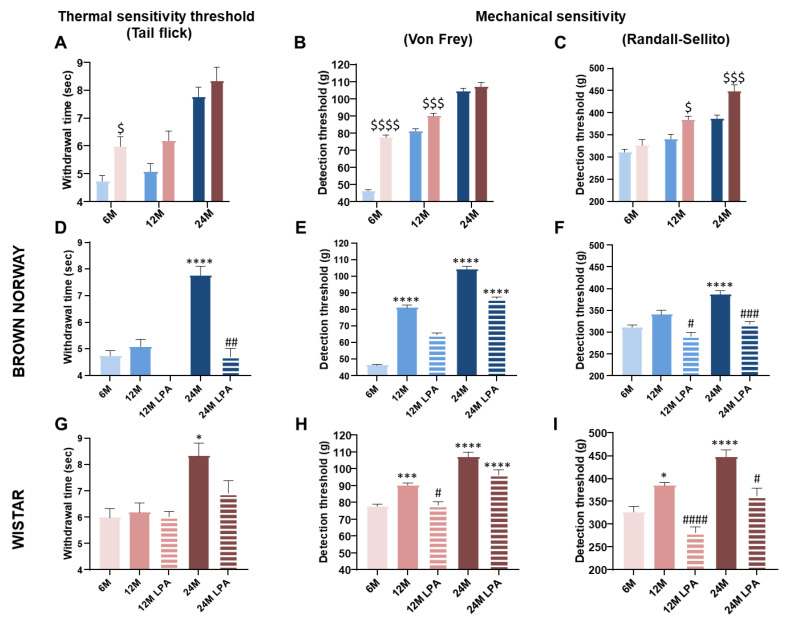
Evolution of the thermal and mechanical skin sensitivity over time with (hatched bars) or without (solid bars) LPA treatment. Thermal sensitivity was assessed using the tail flick test (**A**,**D**,**G**) and mechanical sensitivity with Von Frey (**B**,**E**,**H**) and Randall–Selitto (**C**,**F**,**I**) tests. The results were obtained for both BN (in blue) and Wistar (in pink) strains for 6 month old (6 M), 12 month old (12 M), and 24 month old (24 M) rats previously fed a diet supplemented with α-lipoic acid (0.5%) for 4 weeks (LPA) or not. Error bars represent the standard error of the mean. Statistical analyses were performed revealing significant differences between the two strains for each age group ($ *p* < 0.05; $$$ *p* < 0.001; $$$$ *p* < 0.0001 vs. BN strain) or during skin aging when compared to the 6 month condition (* *p* < 0.05; *** *p* < 0.001; **** *p* < 0.0001) and after LPA treatment compared to age-matched untreated condition (# *p* < 0.05; ## *p* < 0.01; ### *p* < 0.001; #### *p* < 0.0001).

At 6 months, the tail flick (+26%) and Von Frey (+67%) thresholds were higher in Wistar rats compared to BN rats, highlighting a lower sensory sensitivity (Figure 5A,B). In contrast, the Randall–Sellito threshold was not different (around 320 g) between the two strains (Figure 5C). 

A great deal of evidence suggests that sensory thresholds increase with aging, indicating a decline in sensory capacities [38,39,40]. As expected, thermal and mechanical thresholds increased with aging in both strains (Figure 5A–C). However, the deficit in mechanical detection threshold was observed in both strains from 12 months, while the thermal sensitivity was reduced only at 24 months (Figure 5D–I). This growing sensory neuropathy could contribute, at least partially, to the age-related decrease in cutaneous ability to withstand low pressures. Despite some differences between assays, the Wistar strain overall had higher cutaneous thermal and mechanical thresholds compared to the BN strain (Figure 5A–C). This indicates lower skin sensitivity in the Wistar strain regardless of the age, which is consistent with its low resistance to low pressures (lower PIV and positive AUC compared to BN) (Figure 3 and Figure 4). Interestingly, we observed almost similar values between the 12 month old BN and the 6 month old Wistar rats for the Von Frey threshold (Figure 5B) and Randall–Sellito threshold (Figure 5C), as well as for the PIV (Figure 4A) and the positive AUC (Figure 4B). This supports the potential link between skin mechanical sensitivity and skin ability to resist to low pressures.

LPA treatment systematically improved skin nerve sensitivity (Figure 5). In 12 and 24 month old BN rats, a full restoration of tail flick and Randall–Selitto thresholds was observed (Figure 5D,F), whereas the Von Frey test revealed only a partial restoration (Figure 5E). A technical problem occurred for the measurements of the tail flick threshold in LPA-treated BN rats at 12 months. Similar results were obtained in Wistar rats with a full restoration of tail flick threshold at 24 months and Randall–Selitto thresholds at 12 and 24 months (Figure 5G–I), leading to similar thresholds to 6 month old rats. Conversely, a full restoration of the Von Frey threshold was only observed at 12 months (Figure 5E). Overall, LPA treatment either fully or partially restored skin thermal and mechanical nerve sensitivities in both strains at 12 and 24 months.

The beneficial effects of LPA on cutaneous sensory function were insufficient to significantly improve skin resistance capacity (PIV and positive AUC) in 12 and 24 month old BN and Wistar rats. 

In addition to the contribution of sensory nerve endings, PIV also requires intact endothelial function, the NO pathway in particular [27]. Thus, additional experiments were specifically conducted to explore endothelium-dependent and -independent vasodilator capacities of the skin.

### 3.4. LPA Restored the NO Pathway Involved in Endothelial Function in the Wistar Strain

#### 3.4.1. Effect of Aging on Endothelium-Dependent and -Independent Vasodilation

**Figure 6:**
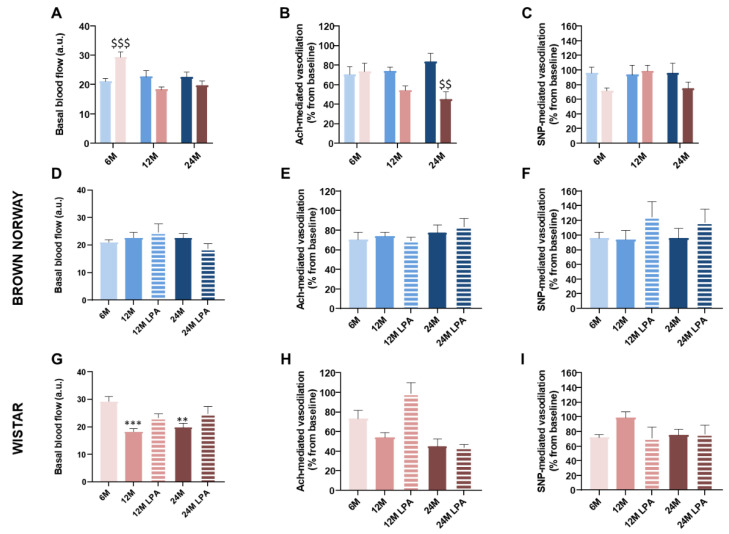
Evolution of the endothelial function over time with (hatched bars) or without (solid bars) LPA treatment. The effect of acetylcholine (Ach) and sodium nitroprusside (SNP) iontophoresis were analyzed in both BN (in blue) and Wistar (in pick) strains for 6 month old (6 M), 12 month old (12 M), and 24 month old (24 M) rats previously fed a diet supplemented with α-lipoic acid (0.5%) for 4 weeks (LPA) or not. A comparison between the two strains was also performed for each parameter (**A**–**C**). In each condition, basal blood flow was measured 1 min prior to Ach or SNP iontophoresis (**A**,**D**,**G**). From the skin blood flow changes, the vasodilation (percentage increase from baseline) was calculated in response to Ach iontophoresis (**B**,**E**,**H**) or to SNP iontophoresis (**C**,**F**,**I**). Error bars represent the standard error of the mean. Statistical analyses were performed revealing significant differences between the two strains for each age group ($$ *p* < 0.01; $$$ *p* < 0.001 vs. BN strain) or during skin aging when compared to the 6 month condition (** *p* < 0.01; *** *p* < 0.001).

Prior to iontophoretic application of Ach or SNP, blood flow value reflects skin perfusion in resting conditions. Basal skin blood flow was significantly higher in Wistar (+39%) compared to BN rats at 6 months, but not at 12 and 24 months (Figure 6A). Indeed, basal blood flow remained unchanged over time in BN rats (Figure 6D), while it significantly decreased at 12 (−38%) and 24 months (−32%) in Wistar rats compared to the 6 month old population (Figure 6G). Interestingly, LPA treatment partially reduced this decrease at 12 and 24 months in Wistar rats (Figure 6G). These results indicate the absence of aging effects on skin perfusion in resting conditions in BN strain. In contrast, an age-related reduction in basal skin perfusion occurred in the Wistar strain that may have been minimized by the LPA treatment.

In healthy skin, local iontophoretic delivery of Ach to the skin is known to induce an endothelium-dependent vasodilation. Indeed, Ach is the most commonly used pharmacological agent to interact with the endothelium and mediates its endothelium-dependent vasodilation via a muscarinic receptor on the endothelial surface. This leads to a rise in intracellular calcium concentration and increases the synthesis and release of endothelial factors via NOS and COX pathways. In contrast, iontophoresis of SNP, an exogenous NO donor, directly stimulates vascular smooth muscle cells inducing an endothelium-independent vasodilation [15,41,42]. 

For both strains, Ach and SNP iontophoresis increased skin blood flow in all conditions, without any significant difference between the two strains at 6 months (Figure 6B,C). 

In BN rats, the vascular responses to Ach and SNP remained unchanged regardless of the age (Figure 6E,F), suggesting that the endothelial function and vascular muscle relaxation are well preserved during skin aging in this strain. In contrast, Ach response tended to decrease progressively (−26% at 12 months and −39% at 24 months) in Wistar rats (Figure 6H), which was not observed for SNP-mediated vasodilation (Figure 6I). 

As expected, LPA treatment changed neither the SNP responses in BN and Wistar rats (Figure 6F,I) nor the responses to Ach in BN rats that were not impacted by aging (Figure 6E). In contrast, LPA treatment strongly increased the Ach-mediated response in Wistar at 12 months (+83% vs. age-matched untreated rats) (Figure 6H), suggesting a complete restoration of the endothelial function that was not observed at 24 months.

#### 3.4.2. Contribution of NOS and COX Pathways over Time

**Figure 7:**
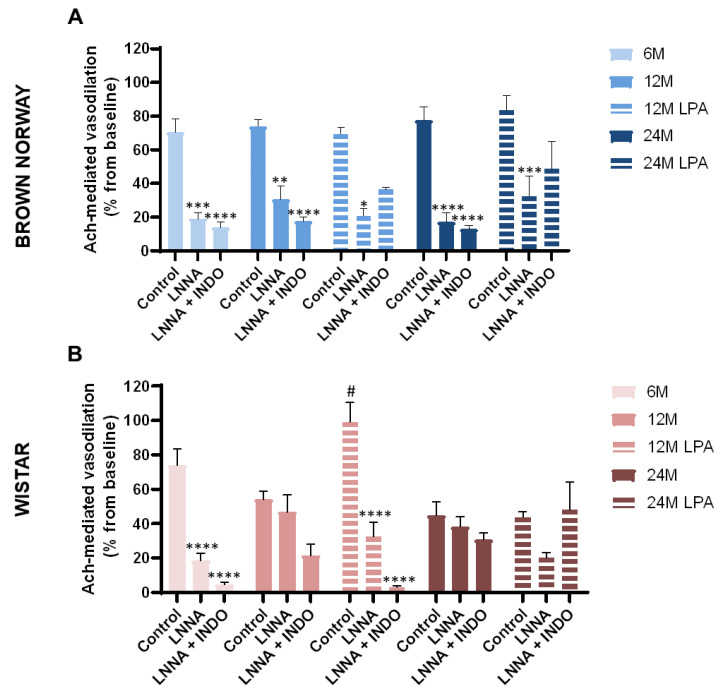
Evolution of the contribution of the NO synthase (NOS) and cyclooxygenase (COX) pathways in the Ach-mediated vasodilation over time with (hatched bars) or without (solid bars) LPA treatment. The effect of an NOS inhibitor (LNNA) in combination or not with a COX inhibitor (INDO) was analyzed on the vascular response to acetylcholine (Ach) iontophoresis in both BN (**A**) and Wistar strains (**B**) for 6 month old (6 M), 12 month old (12 M), and 24 month old (24 M) rats previously fed a diet supplemented with α-lipoic acid (0.5%) for 4 weeks (LPA) or not. In each condition, the blood flow was measured 1 min before and 16 min after iontophoresis. This allowed determining the vasodilation in response to Ach iontophoresis following injection of saline (control) and LNNA alone or in combination with INDO. Results were obtained as a percentage increase from baseline. Error bars represent the standard error of the mean. Statistical analysis was performed revealing significant differences after LPA treatment compared to age-matched untreated condition (# *p* < 0.05) and after LNNA or LNNA + INDO injection when compared to age-matched saline injection as control (* *p* < 0.05; ** *p* < 0.01; *** *p* < 0.001; **** *p* < 0.0001).

As previously observed in untreated rats, Ach-mediated vasodilation was preserved with aging in control BN rats and tended to decrease in control Wistar rats receiving saline (Figure 7A,B).

In BN rats, Ach-mediated vasodilation was abolished by LNNA regardless of the age and not further reduced by LNNA + INDO (Figure 7A). These results suggest that the main pathway involved in this endothelium-dependent vasodilation is the NO pathway, which was well preserved during skin aging in the BN strain. As expected, LPA treatment did not significantly change the effects of the inhibitors.

In Wistar rats, LNNA abolished Ach-mediated vasodilation at 6, but not at 12 and 24 months, showing that the NO pathway was mainly involved at 6 months but strongly impaired by aging in this strain (Figure 7B). In LNNA + INDO conditions, Ach-mediated vasodilation was abolished at 6 months but only reduced at 12 months (−60%) and almost unchanged at 24 months (−32%) (Figure 7B). These results indicate a contribution of the COX pathway in the endothelium-dependent vasodilation in the Wistar strain, in contrast to the BN strain. As for the NO pathway, our results suggest that the COX pathway was also impaired by the aging process in the Wistar strain.

In control Wistar rats receiving saline, LPA treatment significantly increased Ach-mediated vasodilation at 12 months (+83%), but not at 24 months (−2%) (Figure 7B), as previously observed (Figure 6H). In LPA-treated Wistar rats, LNNA reduced Ach-mediated vasodilation (−67% vs. saline at 12 months and −54% at 24 months), showing the contribution of the NO pathway (Figure 7B) that was not involved without LPA at these ages (suggesting beneficial effects of LPA on the NO bioavailability). The combination of LNNA + INDO strongly reduced Ach-mediated vasodilation at 12 months (−97% vs. saline and −91% vs. LNNA only) confirming the contribution of the COX pathway, as previously observed with LPA at 12 months. At 24 months, LNNA + INDO did not reduce Ach-mediated vasodilation, as previously observed without LPA at 24 months, confirming loss of COX pathway at this age. 

## 4. Discussion

The aim of this study was to assess the effect of the antioxidant LPA on skin microcirculation with aging in two different rat strains. We compared a 4 week LPA treatment in a healthy aging model, the BN strain, and in a poorly aging model, the Wistar strain, in middle-aged (12 months) and old (24 months) rats. Our study showed that the age-related reduction in skin ability to resist against low pressures occurred rapidly in Wistar rats, while it was more gradual in BN rats. At 12 months, pressure-induced vasodilation (PIV) was only slightly improved (not significantly) in BN rats and not at all in Wistar rats, when mechanical sensitivity and NO bioavailability were both restored by LPA treatment. In later aging (24 months), the worsening of sensory neuropathy—without aggravation of the endothelial dysfunction—led to the loss of PIV in both strains. At this stage of aging LPA did not improve PIV when sensory neuropathy was only partially restored, as demonstrated by LPA treatment in 24 month old rats.

Aging tended to reduce Ach-dependent vasodilation in Wistar rats without any defect in vascular smooth muscle relaxation. This demonstrates the establishment of an endothelial dysfunction, as already reported in humans [1,43,44,45,46] and animals [4,47,48,49]. More precisely, our results suggested an alteration of the NO pathway in aging Wistar rats. Indeed, Ach-mediated vasodilation became resistant to NOS inhibition in these rats. This reflects an age-related decrease of NO bioavailability in the Wistar strain, as previously reported in old C57BL6 mice [4]. In 12 month old Wistar rats, Ach-mediated vasodilation was abolished under LNNA + INDO, while it was not under LNNA only. This further reduction suggests that the COX pathway contributed to the Ach-dependent vasodilation when rats became resistant to NOS inhibition, as previously reported in aged dyslipidemia mice [49]. However, at 24 months in Wistar rats, Ach-mediated vasodilation was almost unchanged by LNNA + INDO. This suggests that the enhanced contribution of the COX pathway in middle-aged Wistar rats was transient and lost at 24 months. In contrast, BN rats remained sensitive to NOS inhibition regardless of the age, thus strengthening the idea that the NO pathway is mainly involved in this endothelium-dependent vasodilation and well-preserved during skin aging in the BN strain. As BN and Wistar strains are respectively considered “healthy” and “poorly” aging models, this suggests that the COX pathway contributes to endothelial function in a pathological context more than during healthy aging. 

Because vascular function defines age-related end organ damage, it has a great potential to contain health at older age. A correlation between delayed aging and preservation of NO-dependent vasodilation has already been established [50]. Although in vitro study using carotid reported that longevity and endothelial function preservation does not correlate with maximal sensitivity to Ach [51], many studies on various organisms ranging from yeasts to mammals have shown that longevity is correlated to NO pathway preservation [14,52] and eNOS function [53,54]. In BN rats, the NO pathway preservation is, therefore, in accordance with their high longevity. In humans, strong interindividual variations exist during aging. In this context, both “healthy aging” and “poorly aging” models are complementary to better understand the complexity of aging and to progress toward personalized medicine. 

In the current study, LPA (a potent antioxidant with excellent free-radical scavenging capacity) was able to reverse cutaneous endothelial dysfunction in 12 month old Wistar rats. These findings are concordant with the age-related increase in vascular oxidative stress as a consequence of greater production of reactive oxygen species (such as superoxide radical (O_2_^−^), peroxynitrite (ONOO^−^), or hydrogen peroxide (H_2_O_2_)) without a compensatory increase in antioxidant defenses [46,55,56,57,58]. It is largely admitted that the overproduction of reactive oxygen species (ROS) contributes to endothelial dysfunction in aging, both in animals [59] and in humans [56], although ROS may play a compensatory role in endothelial function of the aging microvasculature [47]. Reducing oxidative stress with LPA should subsequently limit NO scavenging and improve NO bioavailability. In 12 month old Wistar rats treated with LPA, the restored Ach-mediated vasodilation was greatly reduced with LNNA, strengthening that LPA effects was largely due to a beneficial effect on NO bioavailability. Similarly, we previously showed that LPA treatment significantly reduced the oxidative stress and was able to preserve endothelial NO availability in the cutaneous microcirculation in short-term diabetic mice [24]. In our previous study LPA treatment was also able to preserve PIV in non-neuropathic diabetic mice [24]. However, in the present study, beneficial effects on endothelial function were not sufficient to improve PIV. All these results indicate that PIV alteration cannot be explained by the sole endothelial alteration in this case. Moreover, BN exhibited reduced PIV with aging without alteration of the NO pathway. 

Indeed, PIV relies not only on vascular function but also requires intact functional integrity of sensory nervous system, especially capsaicin-sensitive nerve endings [11,27] following the activation of acid-sensing ion channel 3 (ASIC3) [13], a mechanoreceptor residing in cutaneous sensory nerve endings. Interestingly, ASIC3 deletion (Asic3 knockout mice) and ASIC3 inhibitors in human and rat skin abolished PIV, without affecting Ach-mediated vasodilation [14], strengthening the crucial role of the sensory nerve endings in PIV. At 6 months, the Wistar strain has an overall lower skin sensitivity than the BN strain (excluding the Randall–Selitto test) associated with lower PIV capacities but equivalent Ach-mediated vasodilation. This reinforces the link between PIV and the cutaneous sensory nerve endings.

With age, a gradual decline affects skin sensory function, with a reduced capacity for thermal and mechanical sensing [1,60,61,62,63] likely due to the widely described reduction in nerve fiber endings [63,64]. Evidence was provided in aged rats for changes in sensory nerve function at both pre- and post-terminal levels [10]. Our results were in concordance with these previous observations, with a progressive age-related decline in cutaneous sensory perception leading to a moderate sensory neuropathy in the middle-aged population and severe sensory neuropathy in the old population. The age-related decline in sensory capacities hardly differed between the two strains even if an early decrease of mechanical sensitivity was observed in Wistar rats according to the Randall–Sellito results.

Interestingly, LPA succeeded in fully restoring nociceptive mechanical and thermal thresholds in the presence of moderate and/or severe sensory neuropathy in BN and Wistar rats. In contrast, LPA failed to restore tactile thresholds in old BN and Wistar rats. Taken together, our results support the efficiency of LPA in reversing (even partially) sensory neuropathy, as previously reported in diabetes [65,66]. It is admitted that large myelinated Aβ nerve fibers primarily transmit nonpainful sensations such as touch (corresponding to Von Frey test), whereas the Aδ/C small nerve fibers are involved in the transduction of pain signals (corresponding to tail flick and Randall–Sellito tests). We, thus, hypothesize that LPA was more efficient on small fibers than on large, myelinated nerve fibers. Moreover, our results confirm the role of oxidative stress into the etiology of cutaneous sensory decline marked by age-related mechanical and thermal hypoalgesia in rats, as largely reported in experimental diabetes and in diabetic subjects [67,68]. 

A moderate sensory neuropathy (without vascular dysfunction) was sufficient to impair the ability of the skin to adapt to localized pressure in BN rats at 12 months leading to the reduction in PIV (−41%), positive AUC (−60%), and crushing pressure (−21%) compared to 6 month old BN rats (although statistical significance was not reached). Associated with endothelial dysfunction, this moderate neuropathy worsened this impairment as observed in Wistar rats. Indeed, PIV (−64%), positive AUC (−85%), and crushing pressure (−69%) were all further reduced in Wistar rats at 12 months compared to 6 months. As a consequence, PIV was significantly lower in Wistar than in BN rats at 12 months. With similar moderate sensory dysfunction, the difference in PIV impairment at 12 months between BN and Wistar could be attributed to endothelial dysfunction (only observed in Wistar rats).

In the presence of severe neuropathy (at 24 months), PIV was almost abolished in the two strains regardless of the vascular capacities. It is worth noting that sensory neuropathy did not worsen the reduction in Ach-mediated vasodilation in Wistar rats, as previously reported in old humans [1]. Therefore, our results showed that the age-related inability of the skin to adapt to localized pressure was related to the severity of the decline of cutaneous sensory capacities rather than to aging-associated endothelial dysfunction. We previously showed that PIV exists in non-neuropathic older adults but is totally absent in old subjects with sensory peripheral neuropathy, while the endothelial dysfunction was comparable between the non-neuropathic and neuropathic older subjects (60–75 years old) [1]. Similar findings have been observed in diabetes. Indeed, PIV was reduced (−8%) in 1 week diabetic mice exhibiting only endothelial dysfunction [24], which was correlated with diabetic patients without neuropathy [69]. The endothelial dysfunction associated with severe neuropathy worsened PIV alteration (−33%) in 8 week diabetic mice [24]. Altogether, these results suggest that the degree of PIV alteration highly depends on the severity of cutaneous sensory decline. Individuals lacking a normal PIV response show an early decrease in cutaneous blood flow to the application of very low pressures [1,69], reflecting a vascular fragility of the skin that increases the risk of ulceration [70]. It is interesting to note that, in a cross-sectional study including 210 older hospitalized adults (mean age 85), Gaubert-Dahan et al. reported that the severity of sensory neuropathy was highly associated with the prevalence and severity of heel pressure ulcer [8]. Sensory neuropathy, thus, appears to be the most critical factor.

Despite obvious beneficial effects on sensory system, LPA failed to fully recover skin capacity to resist against pressures in BN rats at 12 months, even though the PIV (+21%), the positive AUC (+44%), and the crushing pressure (+5%) all increased (without reaching significance). This positive effect was not observed at 24 months, except for the crushing pressure (+56%). Delaying the significant decrease in skin blood flow from baseline for higher crushing pressure suggests a partial beneficial effect of LPA in the BN strain. In contrast, this tendency was not observed at all in middle-aged and old Wistar rats (regarding PIV, positive AUC, and crushing pressure), excluding any beneficial effect of the LPA treatment on the skin resistance to low pressures in the Wistar strain. The failure of LPA in having a benefit in the Wistar strain could have come from the development of the sensory neuropathy in addition to endothelial dysfunction. However, better adjusting LPA treatment modalities could help to improve its beneficial effect. Indeed, a recent study showed that LPA administration in adulthood combined with treatment later in life was more efficient to induce age-induced effects than LPA administration only at old age in male rats [71]. Optimizing doses, duration, and administration modalities (local transcutaneous administration instead of oral administration for example) may improve the effectiveness of LPA treatment, as well as the use of specific preparations favoring LPA stability such as ionic liquid strategy which has recently been reported [21].

## 5. Conclusions

To conclude, our results highlight for the first time an obvious beneficial effect of LPA treatment on age-related cutaneous sensory decline in both the healthy aging BN and the poorly aging Wistar strains (Table 1). Interestingly, LPA treatment was also able to restore NO pathway in the middle-aged Wistar rats, thus improving endothelial function. 

As PIV relies on both skin sensory and endothelial functions, we were expecting an improvement in PIV. However, the beneficial effects of LPA treatment on skin sensory and endothelial functions were only sufficient to partially restore skin resistance to low pressure in BN strain. Better adjusting LPA treatment modalities, alone or in combination with other molecules, may provide a way to improve its effect on skin resistance to pressure and, therefore, on the risk factor to develop skin disorders such as pressure injuries. However, further experiments are needed to define the medicinal doses to be used according to the age and state of health of the subjects, and to better understand the mechanisms by which LPA can improve skin functions.

## Figures and Tables

**Table 1:** Comparison of LPA effects on skin microcirculation and sensory nerve sensitivity in BN and Wistar rats.

	BN Rats	Wistar Rats
**Skin sensory nerve fiber sensitivity**	Alteration of mechanical sensitivity (at 12 and 24 months) and thermal sensitivity (at 24 months only) **Partially or fully restored by LPA**	Alteration of mechanical sensitivity (at 12 and 24 months) and thermal sensitivity (at 24 months only) **Partially or fully restored by LPA**
**Endothelial function** **Endothelial factor contribution**	Preservation over time NOS pathway over time **LPA: no effect**	Decrease from 12 months **Fully restored by LPA** **at 12 months only ** NOS pathway at 6 months COX pathway at 12 months **Beneficial effect on the NOS and COX pathways at 12 months**
**Skin resistance to low pressure**	Gradual decrease over time **Tended to be improved by LPA** **at 12 months only**	Drastic reduction from 12 months **Not improved by LPA**

Endothelial factor contribution and effects of LPA are indicated in grey and orange, respectively. LPA, α-lipoic acid; BN, Brown Norway; NOS, nitric oxide synthase; COX, cyclooxygenase.

## Data Availability

The data presented in this study are available on request from the corresponding author. The data are not publicly available due to ethical restrictions.

## References

[1] Fromy B., Sigaudo-Roussel D., Gaubert-Dahan M.-L., Rousseau P., Abraham P., Benzoni D., Berrut G., Saumet J.L. (2010). Aging-Associated Sensory Neuropathy Alters Pressure-Induced Vasodilation in Humans. J. Invest. Dermatol..

[2] Holowatz L.A., Houghton B.L., Wong B.J., Wilkins B.W., Harding A.W., Kenney W.L., Minson C.T. (2003). Nitric Oxide and Attenuated Reflex Cutaneous Vasodilation in Aged Skin. Am. J. Physiol. Heart Circ. Physiol..

[3] Holowatz L.A., Thompson-Torgerson C., Kenney W.L. (2010). Aging and the Control of Human Skin Blood Flow. Front. Biosci. J. Virtual Libr..

[4] Gaubert M.L., Sigaudo-Roussel D., Tartas M., Berrut G., Saumet J.L., Fromy B. (2007). Endothelium-Derived Hyperpolarizing Factor as an in Vivo Back-up Mechanism in the Cutaneous Microcirculation in Old Mice. J. Physiol..

[5] Bentov I., Reed M.J. (2015). The Effect of Aging on the Cutaneous Microvasculature. Microvasc. Res..

[6] Kenney W.L., Edward F. (2017). Adolph Distinguished Lecture: Skin-Deep Insights into Vascular Aging. J. Appl. Physiol..

[7] De Bengy A.-F., Lamartine J., Sigaudo-Roussel D., Fromy B. (2022). Newborn and Elderly Skin: Two Fragile Skins at Higher Risk of Pressure Injury. Biol. Rev. Camb. Philos. Soc..

[8] Gaubert-Dahan M.-L., Castro-Lionard K., Blanchon M.-A., Fromy B. (2013). Severe Sensory Neuropathy Increases Risk of Heel Pressure Ulcer in Older Adults. J. Am. Geriatr. Soc..

[9] Labeau S.O., Afonso E., Benbenishty J., Blackwood B., Boulanger C., Brett S.J., Calvino-Gunther S., Chaboyer W., Coyer F., Deschepper M. (2021). Prevalence, Associated Factors and Outcomes of Pressure Injuries in Adult Intensive Care Unit Patients: The DecubICUs Study. Intensive Care Med..

[10] Khalil Z., Ralevic V., Bassirat M., Dusting G.J., Helme R.D. (1994). Effects of Ageing on Sensory Nerve Function in Rat Skin. Brain Res..

[11] Fromy B., Abraham P., Saumet J.L. (1998). Non-Nociceptive Capsaicin-Sensitive Nerve Terminal Stimulation Allows for an Original Vasodilatory Reflex in the Human Skin. Brain Res..

[12] Fouchard M., Misery L., Le Garrec R., Sigaudo-Roussel D., Fromy B. (2019). Alteration of Pressure-Induced Vasodilation in Aging and Diabetes, a Neuro-Vascular Damage. Front. Physiol..

[13] Fromy B., Lingueglia E., Sigaudo-Roussel D., Saumet J.L., Lazdunski M. (2012). Asic3 Is a Neuronal Mechanosensor for Pressure-Induced Vasodilation That Protects against Pressure Ulcers. Nat. Med..

[14] Puca A.A., Carrizzo A., Ferrario A., Villa F., Vecchione C. (2012). Endothelial Nitric Oxide Synthase, Vascular Integrity and Human Exceptional Longevity. Immun. Ageing A.

[15] Rossman M.J., LaRocca T.J., Martens C.R., Seals D.R. (2018). Healthy Lifestyle-Based Approaches for Successful Vascular Aging. J. Appl. Physiol..

[16] Seals D.R., Nagy E.E., Moreau K.L. (2019). Aerobic Exercise Training and Vascular Function with Ageing in Healthy Men and Women. J. Physiol..

[17] Beitner H. (2003). Randomized, Placebo-Controlled, Double Blind Study on the Clinical Efficacy of a Cream Containing 5% Alpha-Lipoic Acid Related to Photoageing of Facial Skin. Br. J. Dermatol..

[18] El-Komy M., Shalaby S., Hegazy R., Abdel Hay R., Sherif S., Bendas E. (2017). Assessment of Cubosomal Alpha Lipoic Acid Gel Efficacy for the Aging Face: A Single-Blinded, Placebo-Controlled, Right-Left Comparative Clinical Study. J. Cosmet. Dermatol..

[19] Kubota Y., Musashi M., Nagasawa T., Shimura N., Igarashi R., Yamaguchi Y. (2019). Novel Nanocapsule of α-Lipoic Acid Reveals Pigmentation Improvement: α-Lipoic Acid Stimulates the Proliferation and Differentiation of Keratinocyte in Murine Skin by Topical Application. Exp. Dermatol..

[20] Külkamp-Guerreiro I.C., Souza M.N., Bianchin M.D., Isoppo M., Freitas J.S., Alves J.A., Piovezan A.P., Pohlmann A.R., Guterres S.S. (2013). Evaluation of Lipoic Acid Topical Application on Rats Skin Wound Healing. Acta Cir. Bras..

[21] Zhou Z., Liu C., Wan X., Fang L. (2020). Development of a w/o Emulsion Using Ionic Liquid Strategy for Transdermal Delivery of Anti—Aging Component α—Lipoic Acid: Mechanism of Different Ionic Liquids on Skin Retention and Efficacy Evaluation. Eur. J. Pharm. Sci. Off. J. Eur. Fed. Pharm. Sci..

[22] Bilska A., Włodek L. (2005). Lipoic Acid-the Drug of the Future?. Pharmacol. Rep..

[23] Ho Y.-S., Lai C.-S., Liu H.-I., Ho S.-Y., Tai C., Pan M.-H., Wang Y.-J. (2007). Dihydrolipoic Acid Inhibits Skin Tumor Promotion through Anti-Inflammation and Anti-Oxidation. Biochem. Pharmacol..

[24] Demiot C., Fromy B., Saumet J.L., Sigaudo-Roussel D. (2006). Preservation of Pressure-Induced Cutaneous Vasodilation by Limiting Oxidative Stress in Short-Term Diabetic Mice. Cardiovasc. Res..

[25] Lipman R.D., Chrisp C.E., Hazzard D.G., Bronson R.T. (1996). Pathologic Characterization of Brown Norway, Brown Norway x Fischer 344, and Fischer 344 x Brown Norway Rats with Relation to Age. J. Gerontol. A Biol. Sci. Med. Sci..

[26] Harraz O.F., Jensen L.J. (2020). Aging, Calcium Channel Signaling and Vascular Tone. Mech. Ageing Dev..

[27] Fromy B., Merzeau S., Abraham P., Saumet J.-L. (2000). Mechanisms of the Cutaneous Vasodilator Response to Local External Pressure Application in Rats: Involvement of CGRP, Neurokinins, Prostaglandins and NO. Br. J. Pharmacol..

[28] Fizanne L., Fromy B., Preckel M.-P., Sigaudo-Roussel D., Saumet J.L. (2003). Effect of Isoflurane on Skin-Pressure-Induced Vasodilation. J. Vasc. Res..

[29] Garry A., Sigaudo-Roussel D., Merzeau S., Dumont O., Saumet J.L., Fromy B. (2005). Cellular Mechanisms Underlying Cutaneous Pressure-Induced Vasodilation: In Vivo Involvement of Potassium Channels. Am. J. Physiol. Heart Circ. Physiol..

[30] Deuis J.R., Dvorakova L.S., Vetter I. (2017). Methods Used to Evaluate Pain Behaviors in Rodents. Front. Mol. Neurosci..

[31] Yam M.F., Loh Y.C., Oo C.W., Basir R. (2020). Overview of Neurological Mechanism of Pain Profile Used for Animal “Pain-Like” Behavioral Study with Proposed Analgesic Pathways. Int. J. Mol. Sci..

[32] Sigaudo-Roussel D., Demiot C., Fromy B., Koïtka A., Lefthériotis G., Abraham P., Saumet J.L. (2004). Early Endothelial Dysfunction Severely Impairs Skin Blood Flow Response to Local Pressure Application in Streptozotocin-Induced Diabetic Mice. Diabetes.

[33] Vivancos G.G., Verri W.A., Cunha T.M., Schivo I.R.S., Parada C.A., Cunha F.Q., Ferreira S.H. (2004). An Electronic Pressure-Meter Nociception Paw Test for Rats. Braz. J. Med. Biol. Res. Rev. Bras. Pesqui. Medicas E Biol..

[34] Yeomans D.C., Proudfit H.K. (1996). Nociceptive Responses to High and Low Rates of Noxious Cutaneous Heating Are Mediated by Different Nociceptors in the Rat: Electrophysiological Evidence. Pain.

[35] Nagy J.I., Vincent S.R., Staines W.A., Fibiger H.C., Reisine T.D., Yamamura H.I. (1980). Neurotoxic Action of Capsaicin on Spinal Substance P Neurons. Brain Res..

[36] Nakamura K., Kuntzman R., Maggio A.C., Augulis V., Conney A.H. (1973). Influence of 6-Hydroxydopamine on the Effect of Morphine on the Tail-Flick Latency. Psychopharmacologia.

[37] Le Bars D., Gozariu M., Cadden S.W. (2001). Animal Models of Nociception. Pharmacol. Rev..

[38] Bowden J.L., McNulty P.A. (2013). Age-Related Changes in Cutaneous Sensation in the Healthy Human Hand. Age.

[39] Viseux F.J.F. (2020). The Sensory Role of the Sole of the Foot: Review and Update on Clinical Perspectives. Neurophysiol. Clin. Clin. Neurophysiol..

[40] Taguchi T., Ota H., Matsuda T., Murase S., Mizumura K. (2010). Cutaneous C-Fiber Nociceptor Responses and Nociceptive Behaviors in Aged Sprague-Dawley Rats. Pain.

[41] Morris S.J., Shore A.C. (1996). Skin Blood Flow Responses to the Iontophoresis of Acetylcholine and Sodium Nitroprusside in Man: Possible Mechanisms. J. Physiol..

[42] Melik Z., Zaletel P., Virtic T., Cankar K. (2017). L-Arginine as Dietary Supplement for Improving Microvascular Function. Clin. Hemorheol. Microcirc..

[43] Algotsson A., Nordberg A., Winblad B. (1995). Influence of Age and Gender on Skin Vessel Reactivity to Endothelium-Dependent and Endothelium-Independent Vasodilators Tested with Iontophoresis and a Laser Doppler Perfusion Imager. J. Gerontol. A Biol. Sci. Med. Sci..

[44] Rossi M., Cupisti A., Mariani S., Santoro G., Pentimone F. (2002). Endothelium-Dependent and Endothelium-Independent Skin Vasoreactivity in the Elderly. Aging Clin. Exp. Res..

[45] Tao J., Jin Y., Yang Z., Wang L., Gao X., Lui L., Ma H. (2004). Reduced Arterial Elasticity Is Associated with Endothelial Dysfunction in Persons of Advancing Age: Comparative Study of Noninvasive Pulse Wave Analysis and Laser Doppler Blood Flow Measurement. Am. J. Hypertens..

[46] Seals D.R., Jablonski K.L., Donato A.J. (2011). Aging and Vascular Endothelial Function in Humans. Clin. Sci..

[47] Muller-Delp J.M., Spier S.A., Ramsey M.W., Delp M.D. (2002). Aging Impairs Endothelium-Dependent Vasodilation in Rat Skeletal Muscle Arterioles. Am. J. Physiol. Heart Circ. Physiol..

[48] Woodman C.R., Price E.M., Laughlin M.H. (2003). Selected Contribution: Aging Impairs Nitric Oxide and Prostacyclin Mediation of Endothelium-Dependent Dilation in Soleus Feed Arteries. J. Appl. Physiol..

[49] Gendron M.-E., Thorin-Trescases N., Villeneuve L., Thorin E. (2007). Aging Associated with Mild Dyslipidemia Reveals That COX-2 Preserves Dilation despite Endothelial Dysfunction. Am. J. Physiol. Heart Circ. Physiol..

[50] James M.A., Tullett J., Hemsley A.G., Shore A.C. (2006). Effects of Aging and Hypertension on the Microcirculation. Hypertension.

[51] Labinskyy N., Csiszar A., Orosz Z., Smith K., Rivera A., Buffenstein R., Ungvari Z. (2006). Comparison of Endothelial Function, O2-* and H2O2 Production, and Vascular Oxidative Stress Resistance between the Longest-Living Rodent, the Naked Mole Rat, and Mice. Am. J. Physiol. Heart Circ. Physiol..

[52] Nisoli E., Tonello C., Cardile A., Cozzi V., Bracale R., Tedesco L., Falcone S., Valerio A., Cantoni O., Clementi E. (2005). Calorie Restriction Promotes Mitochondrial Biogenesis by Inducing the Expression of ENOS. Science.

[53] Rexhaj E., Paoloni-Giacobino A., Rimoldi S.F., Fuster D.G., Anderegg M., Somm E., Bouillet E., Allemann Y., Sartori C., Scherrer U. (2013). Mice Generated by in Vitro Fertilization Exhibit Vascular Dysfunction and Shortened Life Span. J. Clin. Invest..

[54] Li W., Mital S., Ojaimi C., Csiszar A., Kaley G., Hintze T.H. (2004). Premature Death and Age-Related Cardiac Dysfunction in Male ENOS-Knockout Mice. J. Mol. Cell. Cardiol..

[55] Muller-Delp J.M., Gurovich A.N., Christou D.D., Leeuwenburgh C. (2012). Redox Balance in the Aging Microcirculation: New Friends, New Foes, and New Clinical Directions. Microcirculation.

[56] Donato A.J., Machin D.R., Lesniewski L.A. (2018). Mechanisms of Dysfunction in the Aging Vasculature and Role in Age-Related Disease. Circ. Res..

[57] Rodríguez-Mañas L., El-Assar M., Vallejo S., López-Dóriga P., Solís J., Petidier R., Montes M., Nevado J., Castro M., Gómez-Guerrero C. (2009). Endothelial Dysfunction in Aged Humans Is Related with Oxidative Stress and Vascular Inflammation. Aging Cell.

[58] Ungvari Z., Kaley G., de Cabo R., Sonntag W.E., Csiszar A. (2010). Mechanisms of Vascular Aging: New Perspectives. J. Gerontol. A Biol. Sci. Med. Sci..

[59] Van der Loo B., Labugger R., Skepper J.N., Bachschmid M., Kilo J., Powell J.M., Palacios-Callender M., Erusalimsky J.D., Quaschning T., Malinski T. (2000). Enhanced Peroxynitrite Formation Is Associated with Vascular Aging. J. Exp. Med..

[60] Decorps J., Saumet J.L., Sommer P., Sigaudo-Roussel D., Fromy B. (2014). Effect of Ageing on Tactile Transduction Processes. Ageing Res. Rev..

[61] Skedung L., El Rawadi C., Arvidsson M., Farcet C., Luengo G.S., Breton L., Rutland M.W. (2018). Mechanisms of Tactile Sensory Deterioration amongst the Elderly. Sci. Rep..

[62] Guergova S., Dufour A. (2011). Thermal Sensitivity in the Elderly: A Review. Ageing Res. Rev..

[63] McIntyre S., Nagi S.S., McGlone F., Olausson H. (2021). The Effects of Ageing on Tactile Function in Humans. Neuroscience.

[64] Namer B. (2010). Age Related Changes in Human C-Fiber Function. Neurosci. Lett..

[65] Vallianou N., Evangelopoulos A., Koutalas P. (2009). Alpha-Lipoic Acid and Diabetic Neuropathy. Rev. Diabet. Stud. RDS.

[66] Ziegler D., Hanefeld M., Ruhnau K.J., Meissner H.P., Lobisch M., Schütte K., Gries F.A. (1995). Treatment of Symptomatic Diabetic Peripheral Neuropathy with the Anti-Oxidant Alpha-Lipoic Acid. A 3-Week Multicentre Randomized Controlled Trial (ALADIN Study). Diabetologia.

[67] Garrett N.E., Malcangio M., Dewhurst M., Tomlinson D.R. (1997). Alpha-Lipoic Acid Corrects Neuropeptide Deficits in Diabetic Rats via Induction of Trophic Support. Neurosci. Lett..

[68] Nickander K.K., McPhee B.R., Low P.A., Tritschler H. (1996). Alpha-Lipoic Acid: Antioxidant Potency against Lipid Peroxidation of Neural Tissues in Vitro and Implications for Diabetic Neuropathy. Free Radic. Biol. Med..

[69] Koïtka A., Abraham P., Bouhanick B., Sigaudo-Roussel D., Demiot C., Saumet J.L. (2004). Impaired Pressure-Induced Vasodilation at the Foot in Young Adults with Type 1 Diabetes. Diabetes.

[70] Fromy B., Josset-Lamaugarny A., Aimond G., Pagnon-Minot A., Marics I., Tattersall G.J., Moqrich A., Sigaudo-Roussel D. (2018). Disruption of TRPV3 Impairs Heat-Evoked Vasodilation and Thermoregulation: A Critical Role of CGRP. J. Invest. Dermatol..

[71] Molz P., de Freitas B.S., Uberti V.H., da Costa K.M., Kist L.W., Bogo M.R., Schröder N. (2021). Effects of Lipoic Acid Supplementation on Age- and Iron-Induced Memory Impairment, Mitochondrial DNA Damage and Antioxidant Responses. Eur. J. Nutr..

